# Molecular analysis of the evolutionary history of endometrial and ovarian carcinoma in Lynch syndrome

**DOI:** 10.1002/ijc.70074

**Published:** 2025-08-07

**Authors:** Anni K. Kauppinen, Alisa P. Olkinuora, Jukka‐Pekka Mecklin, Päivi T. Peltomäki

**Affiliations:** ^1^ Department of Medical and Clinical Genetics, Medicum University of Helsinki Helsinki Finland; ^2^ Department of Education & Research The Wellbeing Services of Central Finland Jyväskylä Finland; ^3^ Department of Sports and Health Sciences Jyväskylä University Jyväskylä Finland; ^4^ HUSLAB Laboratory of Genetics, HUS Diagnostic Center, HUS Helsinki University Hospital Helsinki Finland

**Keywords:** endometrial carcinoma, Lynch syndrome, ovarian carcinoma, panel sequencing, somatic variant

## Abstract

Lynch syndrome (LS) is a prevalent cause of hereditary gynecological cancers. DNA mismatch repair (MMR) defects are important players in LS tumorigenesis, but the developmental steps leading to malignancy are incompletely understood. We undertook a deep sequencing approach with a panel of ~1,000 cancer‐associated genes to detect somatic changes in retrospective specimens from 33 LS carriers who had developed endometrial carcinoma (EC) or ovarian carcinoma (OC). Consecutive samples of atypical endometrial hyperplasia (AH) and EC or OC (64 samples plus blood) were available from a screening period of 15 years (0–15 years). Of carcinomas, all but one (41/42, 98%) were MMR‐deficient by microsatellite instability or immunohistochemical analysis, and 86% (36/42) showed loss of heterozygosity or somatic variants of MMR genes as putative second hits. AH closely resembled EC and OC with respect to MMR deficiency (20/22, 91%) and the presence of second hits (16/22, 73%); moreover, the average tumor mutation burdens and top mutant genes were largely similar in hyperplasia and carcinoma. The proportion of hypermutated tumors (over 10 somatic non‐synonymous mutations per megabase) was 36/42 (86%) among carcinomas and 15/22 (68%) among hyperplasia specimens (statistically non‐significant difference). In individual patients, cancer‐associated genes revealed varying degrees of somatic variant sharing between consecutive specimens of hyperplasia and carcinoma (10/19, 53%), and in some, such variants were detectable in histologically normal endometrium (9/19, 47%) too, one or several years before carcinoma. Our results shed light on the evolutionary trajectories of gynecological cancer development in LS.

## What's New?

Lynch syndrome is a prevalent cause of hereditary gynecological cancers, but the timeline of molecular alterations preceding malignancy remains unclear. By studying consecutive surveillance specimens for up to 15 years before cancer diagnosis, the authors found that atypical endometrial hyperplasia was indistinguishable from endometrial and ovarian carcinoma with respect to mismatch repair deficiency, “second hit” frequency, and somatic mutational profiles. Some variants shared between hyperplasia and carcinoma were also detectable in normal endometrium, suggesting the existence of carcinogenic aberrations long before clinical cancer in Lynch syndrome.

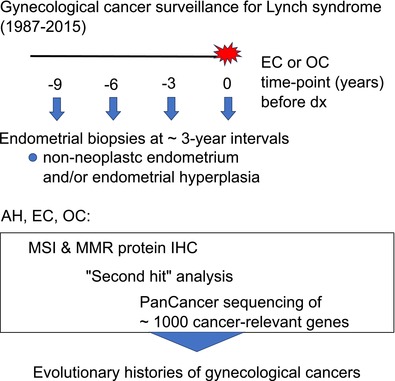

**Abbreviations**
AH: atypical hyperplasia
B: blood
CAH: complex atypical hyperplasia
CCEC: clear cell endometrial carcinoma
CCOC: clear cell ovarian carcinoma
CH: complex hyperplasia without atypia
CxEC: cervical adenocarcinoma
EC: endometrial carcinoma
EEC: endometrioid endometrial carcinoma
EOC: endometrioid ovarian carcinoma
FT: normal fallopian tube
IGV: Integrative Genomics Viewer
IHC: immunohistochemistry
LOH: loss of heterozygosity
LS: Lynch syndrome
MMR: mismatch repair
MSI: microsatellite instability
N: normal
NE: normal endometrium
OC: ovarian carcinoma
SH: simple hyperplasia
T: tumor
TMB: tumor mutational burden
VAF: variant allele frequency

## 1 INTRODUCTION

Endometrial carcinoma (EC) is the most common gynecological malignancy in the Western world, while ovarian carcinoma (OC) is the leading cause of death from a gynecological cancer.^1^ In the average population, the lifetime risks of EC and OC are 1.6% and 1.0%, respectively. In women with Lynch syndrome (LS) carrying pathogenic constitutional variants in the DNA mismatch repair (MMR) genes *MLH1*, *MSH2*, or *MSH6*, the lifetime risks are significantly higher, and the mutated gene greatly affects cancer susceptibility.^2^ Thus, 37%, 49%, and 41% of females carrying pathogenic germline variants of *MLH1*, *MSH2*, and *MSH6*, respectively, may develop EC during their lifetime. The corresponding rates for OC are 11%, 17%, and 11%, respectively.^2^ Deficient MMR is thought to accelerate tumorigenesis in cancer‐prone organs, and the incidence of gynecological malignancies in LS starts to increase after 40 years of age, 10–20 years earlier than average.^3^

Histologically, most ECs from sporadic and LS cases represent type I (estrogen‐dependent, endometrioid) tumors, and stepwise development from endometrial epithelium via endometrial hyperplasia has been proposed.^4^ A minority of ECs belong to the type II category with primarily serous histology. Among epithelial OCs, histopathological, immunohistochemical, and molecular genetic studies have identified five subgroups: high‐grade serous, endometrioid, clear cell, mucinous, and low‐grade serous carcinomas.^5^, ^6^ Endometrioid, clear cell, and mucinous OCs represent type I tumors in analogy to EC, while high‐grade serous OCs represent type II tumors, and neither type I nor type II accurately describes low‐grade serous tumors.^5^ Most OCs from sporadic cases are of high‐grade serous histology, whereas those from LS individuals are predominantly endometrioid or mixed (mucinous/endometrioid/clear cell) types.^7^ The cellular origins and precursors of OC have been under debate for a long time. According to the current understanding, most histological types may be extraovarian in origin.^5^, ^6^ Recent research is now stratifying histological types of EC and OC further into molecular subtypes with prognostic correlations.^6^

Females diagnosed with either EC or OC turn out to have synchronous EC and/or OC in up to 10% of sporadic cases^8^ and 20% of LS cases.^9^ The prognosis of synchronous EC and OC is favorable, which might suggest two primaries rather than metastatic disease. However, molecular studies indicate predominantly shared origins for synchronous gynecological malignancies in both sporadic^10^, ^11^ and LS cases.^12^, ^13^ Molecular attributes of the indolent behavior of synchronous carcinomas await identification.

Recently, sensitive DNA sequencing techniques have detected oncogenic variants in surprisingly high proportions of histologically normal endometria, even from females with no gynecological malignancy.^14^, ^15^ Signs of deficient MMR in non‐neoplastic endometrium, based on microsatellite instability (MSI)^16^, ^17^ or clusters of glands with absent MMR protein,^18^, ^19^ are a specific feature of individuals with LS. The clinical significance of MMR aberrations in histologically normal endometrium (NE) is unclear. Moreover, the precise mechanisms of somatic MMR gene inactivation in non‐neoplastic endometrial glands and endometrial hyperplasia from LS females remain to be determined.

To address some of the open questions that remain, such as the timeline of molecular aberrations before cancer diagnosis, we took advantage of regular gynecological screening of LS individuals^20^ with consecutive endometrial biopsies during the past 15 years. This surveillance provided us with samples from carriers of pathogenic variants of *MLH1* and *MSH2*. We report a frequent occurrence of shared somatic variants between hyperplasia and cancers, sometimes present in non‐neoplastic endometrium as well, raising the scenario that potentially carcinogenic molecular aberrations may be detectable long before clinical cancer in LS.

## 2 MATERIALS AND METHODS

### 2.1 Patients and samples

**FIGURE 1:**
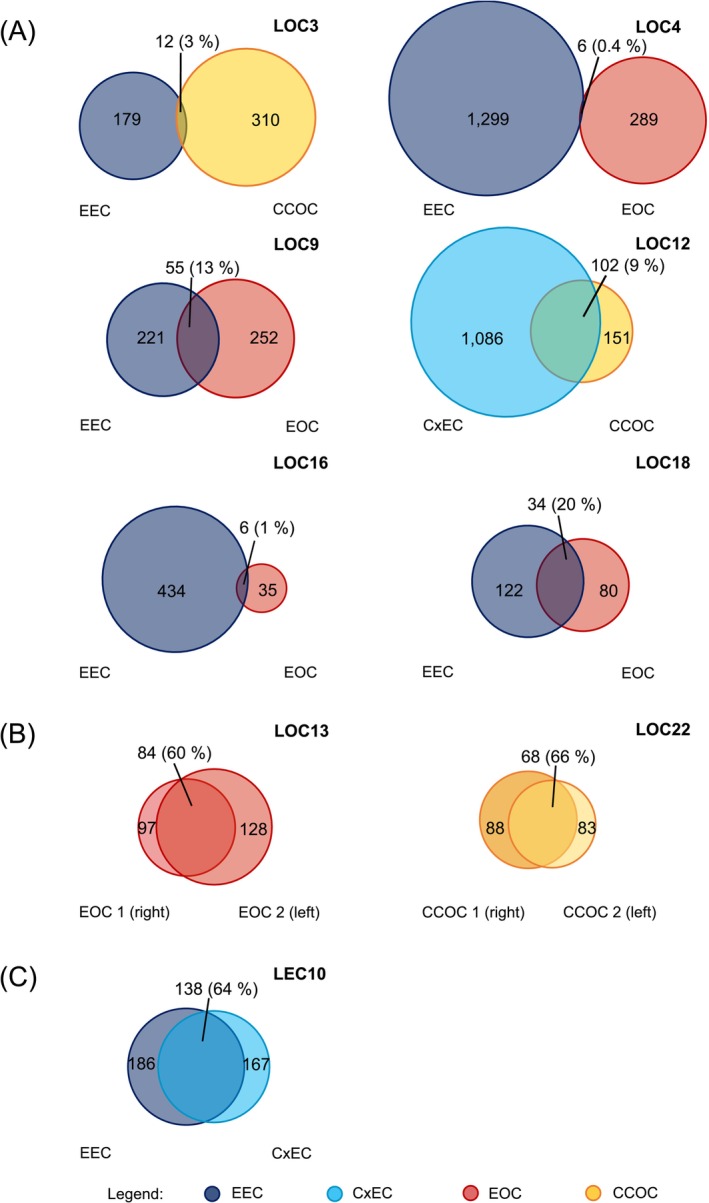
Variant sharing between pairs of synchronous carcinomas shown as Venn diagrams. Pairs of synchronous endometrial carcinoma (EC) and ovarian carcinoma (OC) (A), two OCs (B), and two ECs (C) are depicted. Somatic variants were called against blood in all cases except for LOC9 and LOC16, where normal endometrium was used as a reference in the absence of blood. The percentage of shared somatic variants was calculated according to the following formula: (no. of variants shared by cancers 1 and 2)/[[no. of variants shared by cancers 1 and 2] + [no. of variants unique to cancer 1] + [no. of variants unique to cancer 2]]). CCOC, clear cell ovarian carcinoma; CxEC, cervical adenocarcinoma; EEC, endometrioid endometrial carcinoma; EOC, endometrioid ovarian carcinoma.

The study cohort was ascertained via the nationwide LS Research Registry of Finland from verified LS carriers diagnosed with EC and/or OC; the eligible individuals had been enrolled in regular gynecological surveillance, and the availability of consecutive endometrial biopsy samples together with cancer samples was required.^16^ Tissue specimens consisted of surgical specimens at timepoint 0 and endometrial aspirates at previous timepoints. Formalin‐fixed paraffin‐embedded tissues from different time points up to 15 (mean 2.6) years before carcinoma^16^ were available from 33 LS patients. Normal, hyperplasia, and tumor tissues were separated from a common timepoint sample by manual microdissection.^16^ Among the 33 patients, 28 were carriers of *MLH1* and 5 carriers of *MSH2*, and 26 had EC and 13 had OC (some patients had multiple gynecological malignancies). The average age of gynecological carcinoma diagnosis in this study was 47 years. Among carcinoma patients, 21 had concurrent or earlier endometrial hyperplasia(s). Our endometrial hyperplasia specimens had originally been classified according to the WHO 1994 scheme^21^ into complex hyperplasia without atypia (CH), complex atypical hyperplasia (CAH), and simple hyperplasia (SH) as reported in Niskakoski et al.^16^, ^22^ The new World Health Organization (WHO) classification^23^ recognizes only two categories based on the absence versus presence of atypia. We were unable to adopt the new system to classify the previous CH, since our CH group was molecularly indistinguishable from CAH, yet atypia had not been diagnosed originally and histological materials were unavailable for re‐evaluation. We have, therefore, kept the original WHO 1994 classifications for endometrial hyperplasia in datasets giving sample‐specific results individually for each patient. Statistical calculations, data summaries, and text consider the CAH group alone under “atypical hyperplasia” (AH). We had blood samples from 24 patients. Our carcinoma series included a subgroup of paired carcinomas from nine synchronous cases (Figure 1 below). Based on pathology reports, the paired carcinomas were suspected of having arisen independently, or primary versus metastatic nature was unclear.

### 2.2 MSI and MMR protein expression

DNA was extracted as described by Isola et al.^24^ and investigated for MSI with mononucleotide repeat markers BAT25 and BAT26.^16^, ^22^ Amplified products were separated with ABI3730xl DNA Analyzer. Fragment analysis was performed at the Institute for Molecular Medicine Finland (FIMM) Genomics unit supported by HiLIFE and Biocenter Finland. Samples with at least one unstable repeat marker (deviation of 2 or more nucelotides) were considered as having MSI. MMR protein expression was investigated by immunohistochemistry (IHC) as described previously.^16^, ^22^ MMR deficiency was defined as the presence of MSI or absence of a MMR protein or both.

### 2.3 Twist library preparation and custom panel capture

Gynecological tissue and blood DNAs from our retrospective dataset underwent Pan Cancer panel sequencing with a 6.4 mega base (Mb) design and including about 1000 cancer‐related genes and intronic hot spots.^25^ Next‐Generation Sequencing (NGS) library preparation, sequencing, and sequence analysis were performed by the Institute for Molecular Medicine Finland Technology Centre, University of Helsinki.

Genomic DNA (50 ng) was processed according to Twist Custom Panel EF Multiplex Complete kit (Twist Bioscience, San Francisco, CA, USA) with the following modification: 4 μL of 15 μM Adapters used for ligation were unique dual index (UDI) oligos by IDT (Integrated DNA Technologies, Coralville, IA, USA). Library quantification and quality check were performed using LabChip GX Touch HT High Sensitivity assay (PerkinElmer, Shelton, CT, USA) and Qubit Broad Range DNA Assay (Thermo Fisher Scientific, Waltham, MA, USA). Libraries were pooled to 8‐plex or 10‐plex reactions according to DNA concentration (Qubit). The exome enrichment was performed using Twist custom panel probes (6.4 Mb). The captured library pools were quantified for sequencing using KAPA Library Quantification Kit (KAPA Biosystems, Wilmington, MA, USA) and LabChip GX Touch HT High Sensitivity assay.

Libraries were sequenced on NovaSeq6000 system (Illumina, San Diego, CA, USA) using S4 flow cell and standard workflow (Illumina, San Diego, CA, USA), using the NovaSeq6000 v1.5 reagents. The paired end read length was 101 bp. Panel capture design can be found in Table S1 and performance characteristics in Table S2.

### 2.4 DRAGEN analysis pipeline

Germline variants were called and aligned against the GRCh37 reference genome using the Illumina DRAGEN system (Illumina, San Diego, CA, USA) according to the analysis pipeline v3.9. NGS library preparation, sequencing, and sequence analysis were performed by the Institute for Molecular Medicine Finland Technology Centre, University of Helsinki.

### 2.5 Somatic variant calling

Somatic variants were called using patient‐matched blood‐derived DNA whenever possible, with VarScan2 v2.3.2^26^ using default parameters. Somatic variants with a somatic *p*‐value <.01 were considered significant and used in downstream analyses. When blood was not available, a histologically normal sample (endometrium or fallopian tube) taken at the closest possible time point relative to the neoplastic samples (hyperplasias and carcinomas) was used. Variant impact prediction was done with SnpEff v4.0 with Ensembl v68.^27^ Non‐synonymous somatic variants with VarScan2 *p*‐value <.01 (see above), variant allele frequency (VAF) higher than 5%, and with PASS status from the DRAGEN system were included in the analyses. Tumor mutational burden (TMB) was calculated as the number of significant non‐synonymous somatic variants/Mb (panel size 6.4 Mb), and a tumor was considered hypermutated if it contained more than 10 significant somatic variants per Mb.

Frequently mutant genes (top 20) for each histological subtype of EC and OC were retrieved from the COSMIC database (https://cancer.sanger.ac.uk/cosmic) (Table S3) and the involvement of the same genes was analyzed in our sample series. A set of 136 variants that met our inclusion criteria (see above) underwent additional manual curation by visual inspection on the Integrative Genomics Viewer (IGV). Fifteen variants were deemed false and removed, including 6.7% due to poor quality, 66.7% due to inverted or incorrectly placed reads, 13.3% due to incorrect interpretation, and 13.3% due to >5% VAF in the blood sample (the latter to avoid confusion with possible clonal hematopoiesis). Of note, previously reported top mutant genes associated with clonal hematopoiesis^28^ did not show VAF frequencies above 0.02 in our blood samples.

If VarScan2 analysis revealed somatic variants shared between endometrial hyperplasia and carcinoma, the possible existence of the same variants in histologically normal samples was manually inspected with IGV, and those present in at least five reliable reads and with ≥1% VAF were recorded.

### 2.6 MMR gene second hits

**FIGURE 2:**
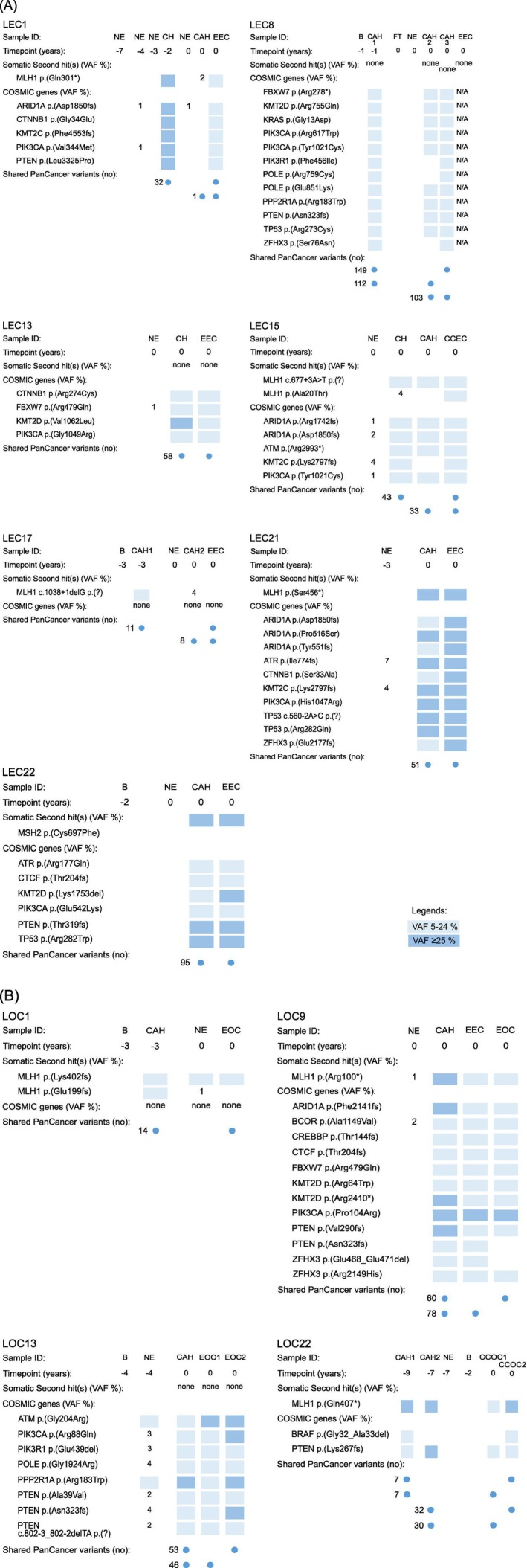
Chronological diagrams of Lynch syndrome cases with shared mismatch repair (MMR) or COSMIC gene variants between hyperplasia and the endpoint lesion(s) endometrial carcinoma (LEC cases, [A]) or ovarian carcinoma (LOC cases, [B]). Timepoint of sample collection is presented as years to carcinoma as endpoint (tp 0). Somatic variants were called against blood in all cases except for LEC1, LEC13, LEC15, LEC21 and LOC9, where normal endometrium was used as a reference in the absence of blood. A somatic variant was listed if it was common between carcinoma and at least one hyperplasia sample and affected any of the top 20 mutated genes listed in the COSMIC database for the carcinoma type in question. For MMR or COSMIC gene variants complying with our regular 5% cut‐off, the density of blue color indicates the range to which each variant's variant allele frequency (VAF) belongs. If an MMR gene second hit variant was discovered from any sample with VAF 5% or higher, the same variant was included in the patient's other samples even if it occurred with lower VAF in these. For variants occurring with VAF below 5% and/or detected in normal endometrium, an exact VAF‐value based on Integrative Genomics Viewer analysis is given. In the bottom part of the figure, numbers next to the dots show the total number of shared variants in PanCancer panel genes in pairwise comparisons between the two tissues identified with the dot (endometrial hyperplasia vs. carcinoma). B, blood; CAH, complex atypical hyperplasia; CCEC, clear cell endometrial carcinoma; CH, complex hyperplasia without atypia; CxEC, cervical adenocarcinoma; EEC, endometrioid endometrial carcinoma; FT, normal fallopian tube; NE, normal endometrium.

Loss of heterozygosity (LOH) analysis was based on constitutionally heterozygous MMR gene variants identified by VarSeq (Golden Helix, version 2.4.0) analysis and verified manually with IGV. For pathogenic constitutional variants of MMR genes, LOH ratio in tumor (T) relative to normal (N) tissue was calculated as (alt:ref)^T^/(alt:ref)^N^ and thresholds for strict and putative LOH were as specified in Ollikainen et al.^29^ Evaluation of somatic MMR gene variants for second hits followed the general somatic variant calling protocol described above, except for relaxed VAF cut‐off in certain cases (see legend to Figure 2).

### 2.7 Clonal relatedness analysis

Analysis of clonal relatedness between pairs of tumor samples based on mutational profiles was conducted by Clonality R package.^30^ We applied the likelihood test. Reference series for EC were derived from cBioPortal Endometrial Carcinoma MSI (MSK, Manning‐Keist et al.^31^) supplemented with non‐synchronous ECs from our present series. Reference series for OC originated from cBioPortal MSK‐IMPACT Clinical Sequencing Cohort (MSK, Zehir et al.^32^) supplemented with non‐synchronous OCs from our present series.

### 2.8 Identification of microsatellite regions in top mutant genes

Microsatellites were identified with the RepeatFinder tool from MANTIS2^33^ program. A region was defined as a microsatellite if the K‐mer length was 1–5 bp, the minimum number of K‐mer repeats was 3, and the minimum number of bases in the region was 5.

### 2.9 Statistical analysis

Statistical analyses were conducted with IBM SPSS Statistics version 29.0.1.0 (171). Data were tested with nonparametric Mann–Whitney *U* when comparing two groups, and nonparametric Kruskal–Wallis followed by Dunn's post hoc test (pairwise comparison) when testing changes between multiple groups. Fisher's exact test was used for two‐level nominal testing, and Bonferroni correction was used when necessary. Two‐tailed *p*‐values <.05 were considered statistically significant.

## 3 RESULTS

### 3.1 MMR status, second hit frequencies, and tumor mutational burdens across sample groups

In this investigation, we aimed to identify key molecular changes and their timing in LS endometrial and ovarian tumorigenesis by studying consecutive samples of endometrial hyperplasia and EC or OC from 33 patients (plus paired blood specimens from 24 of them) under gynecological cancer surveillance for 15 years. Table 1 provides group‐specific summaries of molecular data. Table 2 shows the MMR status, “second hit” status of MMR genes, and TMB for each patient. As evident from Table 2, the average number of tumor (EC, OC, or AH) samples per patient was 1.9 (range 1–4) and most of the samples corresponded to timepoint 0. Variant and other details are available in Table S4.

**TABLE 1:** Clinicopathological data of the study series and summary of second hit and somatic mutational statuses of the sample groups.

Characteristic	EC	OC	EC and OC combined	AH
Number of patients	26	13	33^a^	18
No. of patients with pathogenic germline variant in				
*MLH1*	23	11	28^a^	16
*MSH2*	3	2	5	2
Number of samples for analyses of somatic alterations	27	15	42	22
Proportion of MMR‐deficient samples based on				
MSI (%)	21/27 (78)	15/15 (100)	36/42 (86)	16/21 (76)
IHC (%)	11/11 (100)	10/12 (83)	21/23 (91)	8/8 (100)
MSI or IHC (%)	26/27 (96)	15/15 (100)	41/42 (98)	20/22 (91)
Proportion with detectable second hit (%)				
LOH (No. of samples with LOH or putative LOH out of informative)	6/27 (22)	5/15 (33)	11/42 (26)	6/21 (29)
No. of samples with somatic variant (VAF ≥5%) of the respective MMR gene (out of total)	16/27 (59)	10/15 (67)	26/42 (62)	10/22 (45)
No. of samples with any type of second hit (out of total)	22/27 (81)	14/15 (93)	36/42 (86)	16/22 (73)
No. of non‐synonymous somatic variants *p* < .01, VAF ≥5%, Dragen “PASS”^b^				
Average no. of non‐synonymous somatic variants (range)	253.1 (12–1299)	137.7 (35–310)	211.9 (12–1299)	236.5 (2–928)
Tumor mutational burden (TMB)/Mb, against panel size of 6.4 Mb (range)	39.5 (1.9–203.0)	21.5 (5.5–48.4)	33.1 (1.9–203.0)	37.0 (0.3–145)
	(*n* = 27)	(*n* = 15)	(*n* = 42)	(*n* = 22)
Hypermutated (≥10 non‐synonymous somatic variants/Mb) (%)^b^				
	22/27 (81)	14/15 (93)	36/42 (86)	15/22 (68)

Abbreviations: AH, atypical hyperplasia (all samples in this category represent CAH according to WHO 1994 classification); EC, endometrial carcinoma; OC, ovarian carcinoma; IHC, immunohistochemistry; LOH, loss of heterozygosity; Mb, mega base; MSI; microsatellite instability; VAF, variant allele frequency.

^a^ Includes several patients with multiple lesions, which explains why numbers in left‐hand columns do not add up.

^b^ Against blood or if not available, normal endometrium/normal fallopian tube.

**TABLE 2:** MMR, second hit, and hypermutability statuses patient by patient.

Case ID_Histology	Predisposing gene	Timepoint (years)	MMR Status	MMR gene LOH^a^	Somatic second hit variant^b^ (VAF %)	TMB^c^
LEC1_EEC	*MLH1*	0	dMMR	No	*MLH1* nonsense variant (7%)	6.1^d^
LEC1_CAH	*MLH1*	0	pMMR	No	[*MLH1* nonsense variant (2%)]	0.3^d^
LEC2_EEC	*MSH2*	0	dMMR	No	*MSH2* splice variant (24%)	**16.6**
LEC3_EEC	*MLH1*	0	dMMR	No	*MLH1* splice variant (17%)	**11.6**
LEC4_EEC	*MLH1*	0	dMMR	No	*MLH1* frameshift variant (27%)	**17.0**
LEC5_EEC	*MLH1*	0	dMMR	No	*MLH1* in‐frame deletion variant (28%)	**13.6**
LEC6_CCEC1	*MLH1*	−1	dMMR	No	*MLH1* missense variant (16%)	**12.5**
LEC6_CCEC2	*MLH1*	0	dMMR	No	*MLH1* missense variant (20%)	**14.7**
LEC8_CAH1	*MLH1*	‐1	dMMR	LOH	None	**31.1**
LEC8_CAH2	*MLH1*	0	dMMR	pLOH	None	**18.4**
LEC8_CAH3	*MLH1*	0	dMMR	No	*mlh1* splice variant (5%)	**141.6**
LEC9_CAH	*MLH1*	−3	dMMR	N/A	None	0.8^d^
LEC9_EEC	*MLH1*	0	dMMR	pLOH	None	**14.5** ^d^
LEC10_EEC	*MLH1*	0	dMMR	pLOH	None	**29.1**
LEC10_CxEC	*MLH1*	0	dMMR	pLOH	None	**26.1**
LEC11_CAH	*MLH1*	0	dMMR	pLOH	None	**145.0**
LEC11_EEC	*MLH1*	0	dMMR	No	*MLH1* frameshift variant (14%)	**68.1**
LEC12_CAH	*MLH1*	0	dMMR	No	[*MLH1* nonsense variant (3%)]	**14.5**
LEC12_EEC	*MLH1*	0	dMMR	No	[*MLH1* nonsense variant (4%)]	**23.1**
LEC13_EEC	*MSH2*	0	dMMR	pLOH	None	9.2^d^
LEC14_CAH	*MLH1*	0	pMMR	No	None	0.9
LEC14_EEC	*MLH1*	0	pMMR	No	None	9.5
LEC15_CCEC	*MLH1*	0	dMMR	No	*MLH1* splice variant (20%), *MLH1* missense variant(19%)	**13.6** ^d^
LEC15_CAH	*MLH1*	0	dMMR	No	*MLH1* splice variant (14%)	8.6^d^
LEC16_EEC	*MLH1*	0	dMMR	LOH	None	**36.9** ^d^
LEC16_CAH	*MLH1*	0	dMMR	LOH	None	**15.9** ^d^
LEC17_CAH1	*MLH1*	−3	dMMR	No	*MLH1* splice variant (7%)	4.7
LEC17_EEC	*MLH1*	0	dMMR	No	None	4.4
LEC17_CAH2	*MLH1*	0	dMMR	No	[*MLH1* splice variant (4%)]	9.4
LEC19_EEC	*MLH1*	0	dMMR	No	*MLH1* frameshift variant (28%), *MLH1* missense variant (12%)	**173.1**
LEC21_CAH	*MLH1*	0	dMMR	No	*MLH1* frameshift variant (27%)	**13.4** ^d^
LEC21_EEC	*MLH1*	0	dMMR	No	*MLH1* frameshift variant (35%)	**12.8** ^d^
LEC22_CAH	*MSH2*	0	dMMR	No	*MSH2* missense variant (30%)	**28.0**
LEC22_EEC	*MSH2*	0	dMMR	No	*MSH2* missense variant (29%)	**31.3**
LEC24_EEC	*MLH1*	0	dMMR	No	None	1.9
LOC1_CAH	*MLH1*	−3	dMMR	No	*MLH1* frameshift variant (6%), *MLH1* frameshift variant (9%)	**16.3**
LOC1_EOC	*MLH1*	0	dMMR	No	*MLH1* frameshift variant (5%)	**16.4**
LOC3_EEC	*MLH1*	0	dMMR	No	*MLH1* frameshift variant (20%)	**28.0**
LOC3_CCOC	*MLH1*	0	dMMR	No	*MLH1* frameshift variant (6%)	**48.4**
LOC4_EOC	*MLH1*	0	dMMR	No	*MLH1* missense variant (35%)	**45.2**
LOC4_EEC	*MLH1*	0	dMMR	pLOH	None	**203.0**
LOC5_EOC	*MSH2*	0	dMMR	No	*MSH2* nonsense variant (8%)	**21.4**
LOC5_CAH	*MSH2*	0	dMMR	No	*MSH2* nonsense variant (6%)	**10.0**
LOC9_EOC	*MLH1*	0	dMMR	No	*MLH1* nonsense variant (24%)	**39.4** ^d^
LOC9_EEC	*MLH1*	0	dMMR	No	*MLH1* nonsense variant (21%)	**34.5** ^d^
LOC9_CAH	*MLH1*	0	dMMR	No	*MLH1* nonsense variant (26%)	**75.2** ^d^
LOC12_CCOC	*MLH1*	0	dMMR	No	*MLH1* nonsense variant (21%)	**23.6**
LOC12_CxEC	*MLH1*	0	dMMR	No	*MLH1* nonsense variant (8%)	**169.7**
LOC13_EOC1	*MLH1*	0	dMMR	LOH	None	**15.2**
LOC13_EOC2	*MLH1*	0	dMMR	LOH	None	**20.0**
LOC13_CAH	*MLH1*	0	dMMR	LOH	None	**144.1**
LOC16_EOC	*MLH1*	0	dMMR	No	None	5.5^d^
LOC16_EEC	*MLH1*	0	dMMR	No	*MLH1* missense variant (16%)	**67.8** ^d^
LOC16_CAH	*MLH1*	0	dMMR	No	None	**13.0** ^d^
LOC17_EOC	*MLH1*	0	dMMR	No	*MLH1* frameshift variant+splice variant (30%)	**14.8**
LOC18_EOC	*MLH1*	0	dMMR	LOH	None	**12.5**
LOC18_EEC	*MLH1*	0	dMMR	LOH	None	**19.1**
LOC18_CAH	*MLH1*	0	dMMR	LOH	None	**98.0**
LOC20_EOC	*MSH2*	0	dMMR	LOH	None	**18.6**
LOC21_CCOC	*MLH1*	0	dMMR	LOH	*MLH1* missense variant (28%)	**15** ^d^
LOC22_CAH1	*MLH1*	−9	dMMR	No	*MLH1* nonsense variant (44%)	**14.1**
LOC22_CAH2	*MLH1*	−7	dMMR	No	*MLH1* nonsense variant (20%)	9.8
LOC22_CCOC1	*MLH1*	0	dMMR	No	*MLH1* nonsense variant (6%)	**13.8**
LOC22_CCOC2	*MLH1*	0	dMMR	No	*MLH1* nonsense variant (24%)	**13.0**

Abbreviations: CAH, complex atypical hyperplasia (corresponds to AH, atypical hyperplasia); CCEC, clear cell endometrial carcinoma; CCOC, clear cell ovarian carcinoma; CxEC, cervical adenocarcinoma; dMMR, MMR‐deficient (abnormal result from MSI or IHC analysis or both); EEC, Endometrioid Endometrial Carcinoma; EOC, Endometrioid Ovarian Carcinoma; LEC, Lynch syndrome Endometrial Carcinoma patient id; LOC, Lynch syndrome Ovarian Carcinoma patient id; LOH, loss of heterozygosity; MMR, mismatch repair; pMMR, MMR‐proficient; TMB, tumor mutational burden; VAF, variant allele frequency.

^a^ LOH was recorded if it affected the wild type allele. pLOH denotes putative LOH (see Section 2).

^b^ For details, see Table S4. Variants with VAF below 5% are in brackets.

^c^ Hypermutable samples (TMB ≥10 non‐synonymous somatic variants/mega base) are in bold.

^d^ TMBs calculated against normal endometrium or normal fallopian tube, since blood was not available.

Based on MSI analysis, IHC analysis, or both, MMR was deficient in 91% (20/22) of atypical hyperplasia and 98% (41/42) of carcinoma samples. The frequency of MMR deficiency did not significantly differ between hyperplasia and carcinomas (Table 1). While this complies with the concept that atypical endometrial hyperplasia (AH) is a precursor lesion to carcinoma,^16^, ^34^ it does not rule out alternative routes of tumorigenesis (see Section 4).

A detectable second hit (LOH or a non‐synonymous somatic nucleotide variant) for inactivation of the MMR gene in question was present in 73% (16/22) and 86% (36/42) of hyperplasia and carcinoma samples, respectively (Table 1). Again, carcinomas and atypical hyperplasia showed no statistically significant difference relative to each other. Using our standard VAF cut‐off of 5%, 16/20 (80%) of atypical hyperplasia specimens that were MMR‐deficient had a second hit as opposed to 0/2 of MMR‐proficient cases (*p* = .065 by Fisher's exact test) (Table 2). The single MMR‐proficient carcinoma (LEC14) did not reveal a detectable second hit. LOH and somatic variants were mutually exclusive as second hits: of 17 EC, OC, and atypical hyperplasia samples with LOH or pLOH, only one (clear cell OC of LOC21) showed a somatic MMR gene variant, too, and of 36 samples with somatic second hit variants with VAF ≥5%, only LOC21 showed LOH, too (*p* < .001) (Table 2).

Based on significant non‐synonymous somatic variants (Table S5), TMB, counted as total somatic mutational load divided by the size of the panel (6.4 Mb), exceeded 10 (i.e., indicated hypermutated state) in 68% (15/22) of atypical hyperplasia and 86% (36/42) of carcinoma samples (Table 1). The difference between hyperplasia and carcinoma was statistically non‐significant.

### 3.2 Synchronous carcinomas: primary or metastatic disease?

Our series included nine cases with synchronous carcinomas with samples available from both carcinomas of each pair. We used the degree of variant sharing between the paired carcinomas (Figure 1) for evaluating if the tumors represented two primaries or if one was a metastasis of another. The percentages of variant sharing ranged from 0.4% (LOC4) to 66% (LOC22). Cases with two synchronous ECs (Figure 1C) or OCs (Figure 1B) shared more somatic variants between the paired carcinomas (60%–66%) than cases with an EC plus OC combination (0.4%–20%) (Figure 1A). We interpret our variant sharing data to suggest that in at least seven out of nine cases (78%), the paired carcinomas were likely to have a shared origin. The minimal degree of variant sharing between the paired carcinomas of LOC3 and LOC4 implies that these pairs may comprise two primary tumors (arguing against our earlier interpretation mainly derived from IHC and methylation data for these cases^12^). Variant sharing between the paired EC and OC of LOC16 was low, too, but shared variants comprised 17% (6/35) of all variants present in the OC (Figure 1A), supporting a common origin. Clonality R package (see Section 2) confirmed our interpretations by identifying LOC4 as the only case likely to have two primaries (*p* = .657 suggested that the null hypothesis of two primaries could not be rejected). Our conclusion of most of the paired synchronous carcinomas having shared origins complies with observations from sporadic synchronous cases.^10^, ^11^, ^35^

### 3.3 Molecular characteristics of endometrial hyperplasia and relationship to carcinoma

Counting all non‐synonymous somatic variants fulfilling our selection criteria (VarScan2 *p* < .01), the overall TMB of atypical hyperplasia (*n* = 22) ranged from 0.3 to 145.0/Mb (*average* 37.0) per sample (Table 1). Atypical hyperplasia shared an average of 27.3 (*range* 1–95) somatic variants with their paired carcinomas, and the average percentage of somatic variants shared with the associated carcinoma(s) (out of all somatic variants detected in each hyperplasia sample) was 25.7% (*range* 1.4%–66.7%). The degree of variant sharing did not differ relative to EC versus OC as the endpoint lesion.

For a closer look at the evolutionary histories of gynecological carcinomas in LS, we constructed chronological diagrams of somatic variants shared by consecutive samples of endometrial hyperplasia and the endpoint lesions EC or OC for each patient. We focused on the sharing of somatic “second hit” variants of MMR genes, somatic variants of COSMIC driver genes linked to cancer histology (please see Section 2), and somatic variants of other cancer‐relevant genes included in our PanCancer panel. For COSMIC variants to be displayed in the diagrams, we required that a variant was common between carcinoma and at least one hyperplasia sample. For other than COSMIC genes, variant sharing between carcinoma and two hyperplasia samples was required. Figures 2A,B and S1 depict cases in which shared variants between hyperplasia and carcinoma existed. In the event of variant sharing between endometrial hyperplasia and carcinoma, normal endometrial samples, too, were investigated for the possible presence of the same variants.

As evident from Tables 1 and 2, all cases of atypical hyperplasia except two (LEC1 and LEC14) out of 22 (91%) were MMR‐deficient based on MSI or IHC analysis, or both. In LEC8, LOH was discovered 1 year (Figure 2A), and in LEC17 (Figure 2A) and LOC1 (Figure 2B), somatic second hit variants were found 3 years before carcinoma diagnosis. In LOC22, a somatic *MLH1* p.(Gln407*) variant was present in high allele frequencies (44% and 20%, respectively) in CAHs nine and 7 years before OCs that also had this variant (Table 2; Figure 2B).

As examples of shared COSMIC gene variants between hyperplasia and carcinoma, CAH of LEC22 showed a *PIK3CA* p.(Glu542Lys) variant that has been linked to progression into EC,^36^ and synchronous hyperplasia and EC samples from LEC15 shared *ARID1A*, *ATM*, and *PIK3CA* variants (Figure 2A). *PTEN* frameshift variants occurred in CAH from LEC8 1 year (Figure 2A) and that from LOC22 9 and 7 years before carcinoma(s) (Figure 2B). LEC15, LEC17, and LOC22 illustrate cases with shared somatic variants in non‐COSMIC genes in addition to COSMIC variants (Figure S1).

It was not uncommon to detect variants shared between hyperplasia and carcinoma in NE as well (Figures 2A,B and S1). For example, normal endometria from LEC1 (Figure 2A), LEC21 (Figure 2A), and LOC13 (Figure 2B), respectively, showed COSMIC driver gene variants 3, 3, and 4 years before the same variants were detected in EC or OC. Apart from COSMIC gene variants, LEC15 revealed extensive sharing of non‐COSMIC gene variants between specimens of NE, hyperplasia, and EC (Figure S1).

### 3.4 Top mutant genes

**FIGURE 3:**
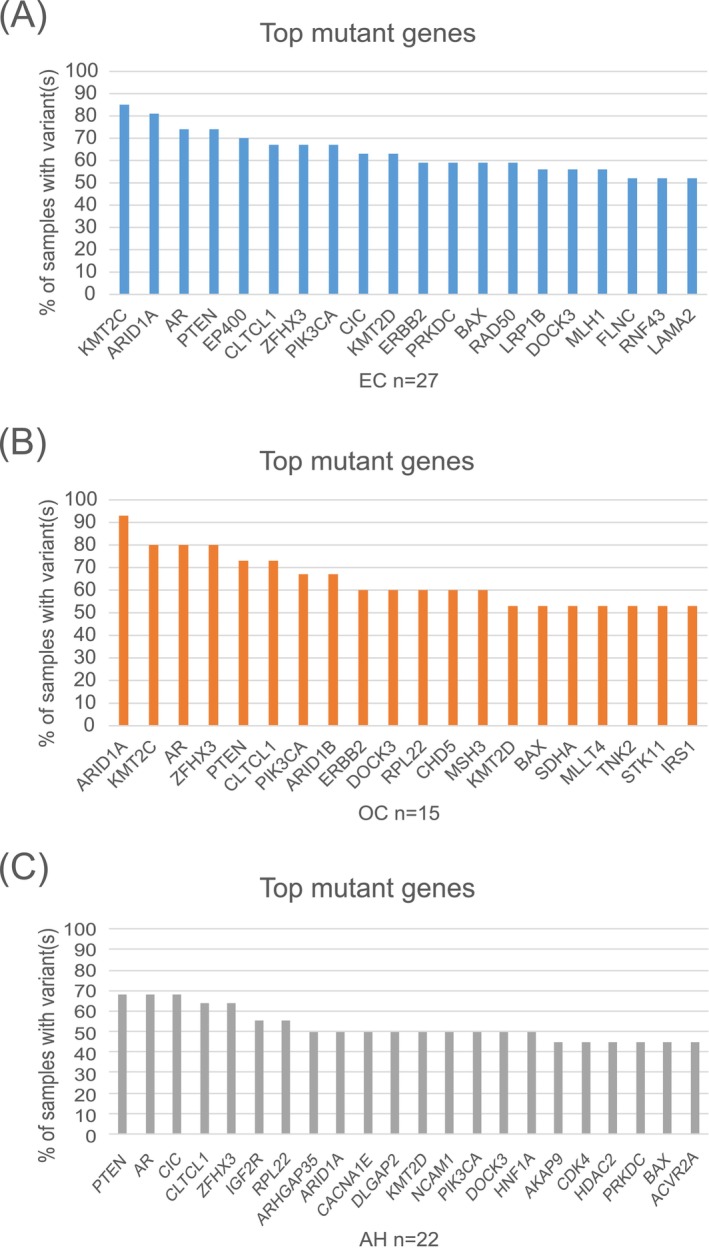
Top mutant genes with variant allele frequencys above 5% in endometrial carcinoma (EC) (A), ovarian carcinoma (OC) (B), and atypical hyperplasia (C). Genes having somatic mutations in over 50% of carcinomas (A and B) and in over 40% of hyperplasia samples (C) are shown. AH, atypical endometrial hyperplasia.

Using VAF 5% as the cut‐off, the top mutant genes among ECs (*n* = 27) were *KTM2C* (85%) and *ARID1A* (81%), followed by *PTEN* and *AR* (74%) (Figure 3A). The most frequently affected gene in OC (*n* = 15) was *ARID1A* (93%), followed by *KTM2C*, *AR*, and *ZFHX3* (80%) (Figure 3B). Among AH (*n* = 22), *PTEN* ranked the highest together with *AR* and *CIC* being mutant in 68% of specimens (Figure 3C). Many top mutant genes, including *ARID1A* and *PTEN*, persisted when VAF 25% was set as a cut‐off (Figure S2). As evident from Figures 3 and S2, there was a significant overlap in top mutant genes between EC, OC, and endometrial hyperplasia. This observation implies shared features in the pathogenesis of EC and OC, which is not surprising since non‐serous (type I) histology predominated among our ECs and OCs as typical of LS in general.^7^ Reflecting MMR deficiency, the relative proportions of frameshift variants among all variants (Table S5) were high for many top mutant genes, including *KMT2C* (36% and 69% for EC and OC, respectively), *ARID1A* (63%, 78%, and 54% for EC, OC, and hyperplasia, respectively), and *PTEN* (54%, 33%, and 59% for EC, OC, and hyperplasia, respectively). Analysis of coding microsatellite repeats (see Section 2) in top mutant genes shared by EC, OC, and AH showed that *BAX* (91%), *AR* (79%) and *ARID1A* (66%) had the highest and *CLTCL1* (2%) and *PIK3CA* (0%) the lowest proportion of variants affecting microsatellite regions.

## 4 DISCUSSION

Recent advances in molecular alterations occurring in EC and OC have led to improved disease classifications for prognostic, predictive, and therapeutic purposes.^6^ However, limited availability of biological specimens from the time before cancer hampers accurate definition of the evolutionary histories leading to EC and/or OC. Our investigation utilized regular gynecological screening of LS individuals as a source of consecutive tissue specimens taken up to 15 years before EC or OC diagnosis. We found that AH revealed striking similarity to ECs and OCs in terms of MMR deficiency, presence of “second hits” in MMR genes, TMBs, and top mutant genes. Interestingly, some of the variants shared between hyperplasia and carcinoma were detectable in NE, too, simultaneously with or before their occurrence in the hyperplasia or carcinoma. Our results offer new insights into endometrial and ovarian tumorigenesis in LS and beyond.

In LS, one allele of *MLH1*, *MSH2*, *MSH6*, or *PMS2* is mutant in every cell and causes cancer predisposition, but tumors usually do not develop until a “second hit” inactivates the remaining wild‐type allele in a cancer‐prone target tissue, such as colonic mucosa or uterine endometrium. Surprisingly, an average of one crypt per 1 cm^2^ was recently found to have extinct MMR protein expression and MSI in non‐neoplastic colonic mucosa from LS individuals.^37^ Glands with absent MMR protein implying biallelic MMR gene inactivation may be even more prevalent in non‐neoplastic endometrium of LS carriers.^19^ It is possible that such MMR‐deficient fields may induce tumor development, at least when combined with other oncogenic events,^19^, ^37^ in analogy to LS colorectal tumorigenesis from MMR‐deficient crypt foci, with or without visible precursor lesions.^38^ In our series, 91% (20/22) of atypical hyperplasia and 98% (41/42) of carcinoma samples were MMR‐deficient, and 73% (16/22) and 86% (36/42) had second hits (Table 1) complying with the two‐hit model of tumorigenesis.

According to epidemiological studies, up to 20%–40% of females with atypical hyperplasia tend to develop EC in a long‐term follow‐up.^34^, ^39^ A mean interval of 6 years between the diagnosis of atypical hyperplasia and clinically manifest EC was reported^40^; the length of this interval in the LS context is unknown. While an increased EC risk in patients with atypical hyperplasia is indisputable, the clonal relationship between the hyperplastic lesions and associated EC or OC is poorly understood. Li et al.^41^ used exome sequencing to examine 30 pairs of newly diagnosed atypical hyperplasia and EC. The MSI‐high subset of atypical hyperplasias (16.7%) showed a high concordance with the MSI status of the paired ECs, leading the authors to conclude that MMR defects represent early events in EC development. With smooth muscle DNA from the patients as reference, the average number of somatic non‐synonymous variants in atypical hyperplasias with MSI‐high was 596 (*range* 170–953), which was comparable to ECs with MSI‐high (*average* 632, *range* 269–1103). Interestingly, the percentage of shared somatic variants between atypical hyperplasias and their paired ECs was significantly lower for MSI‐high than microsatellite‐stable pairs (19% vs. 41%, *p* = .029).^41^ This observation could imply a higher propensity for multifocal origin for the atypical hyperplasia‐EC pairs with MSI, in agreement with observations from sporadic colorectal adenoma‐carcinoma pairs.^42^ As an overall conclusion from their investigation, Li et al.^41^ proposed that some atypical hyperplasia lesions are immediate precursors of ECs, whereas in other cases the two lesions diverge early or arise independently.

In our LS cohort, all but two cases of atypical hyperplasia, and all carcinomas except one, were MMR‐deficient (Table 1). We screened our samples with a panel of ~1000 cancer‐associated genes, and the average number of non‐synonymous somatic variants against blood or, if not available, NE/normal fallopian tube (FT) in atypical hyperplasia (236.5, corresponding to TMB of 37.0/Mb) was very similar to that in EC (253.1, corresponding to TMB of 39.5/Mb) in agreement with Li et al.^41^ The average number of non‐synonymous variants in OC was somewhat lower (137.7, corresponding to TMB of 21.5/Mb) (statistically non‐significant difference). To investigate if the time point of diagnosis influenced somatic mutational loads, we stratified the hyperplasia samples into those occurring concurrently with EC or OC (16 samples) and those preceding EC or OC (six samples, interval to cancer 1–9 years). The average number of somatic variants was 294.5 (*range* 2–928) in the former group versus 81.8 (*range* 5–199) in the latter group, corresponding to TMBs of 46.0/Mb versus 12.8/Mb (statistically non‐significant difference). Longitudinal case‐by‐case analyses suggested sequential trajectories in, for example, LOC22, in which a somatic second hit of *MLH1* and somatic variants in several cancer‐associated genes were shared between the endpoint carcinomas and atypical hyperplasia samples from the time of 9 and 7 years before (Figure 2B). While we lack information on the detailed medical histories of our patients, non‐surgical treatment of atypical hyperplasia typically consists of local or systemic progestin,^43^ and it is possible that such treatment results in incomplete eradication of neoplastic clone(s), allowing tumorigenesis to continue. In cases other than those shown in Figure 2 (10/21 patients), no somatic variants in COSMIC driver genes were shared between endometrial hyperplasia and associated carcinoma, suggesting divergent routes of tumorigenesis.

Occasional variants shared between hyperplasia and carcinoma existed in NE as well (Figures 2A,B and S1). There were examples of shared COSMIC gene variants whose frequencies increased from NE towards hyperplasia or carcinoma (e.g., LEC21 and LOC13 in Figure 2A,B, respectively), which may imply a role in tumorigenesis. However, somatic driver variants are surprisingly frequent among non‐neoplastic endometrial samples^14^, ^17^ or glands^15^ in even average females with no earlier or concurrent gynecological malignancy, and appropriate caution is necessary when interpreting the biological or clinical significance of such alterations.

Previous sequencing studies of paired atypical hyperplasia and EC samples have established a frequent involvement of the PI3K pathway (e.g., *PTEN* and *PIK3CA*) and SWI/SNF complex (e.g., *ARID1A*) genes in both types of lesions.^41^, ^44^, ^45^ Most samples investigated so far have had proficient MMR, and available information on MMR‐deficient lesions, especially endometrial hyperplasia, is limited. In our series of predominantly MMR‐deficient hyperplasia and carcinoma samples, PI3K and SWI/SNF complex genes were among the top mutant genes (Figures 3 and S2), thus complying with previous observations from MMR‐proficient cases. Besides chromatin remodeler genes such as *ARID1A*, other epigenetic regulatory genes (e.g., lysine methyltransferase genes *KTM2C* and *KTM2D*) were often affected by somatic variants (Figures 3 and S2) resembling our previous findings from LS‐associated colorectal tumors.^46^ As a special feature of our top mutant genes (Figures 3 and S2), many (e.g., *ACVR2A*, *BAX*, *HNF1A*, *MSH3*, *PRKDC*, *PTEN*, and *RPL22*) contain repeat tracts as known targets for MSI.^47^, ^48^, ^49^

Consecutive tissue samples from gynecological screening constituted an important strength of this investigation. Our study design was retrospective and included only LS patients who had developed EC or OC. We did not investigate corresponding samples from LS patients who were cancer‐free (such samples were not available), which restricts interpretations of the carcinogenic potential of alterations observed in non‐neoplastic or preneoplastic tissues. Moreover, we lacked access to detailed medical records of our patients. OCs from our series were predominantly of endometrioid or clear cell histology, and these subtypes are thought to arise from endometriosis.^6^ While we cannot exclude endometriosis as a possible inductor of somatic variants in non‐neoplastic endometria from our individual patients, endometriosis is unlikely to be a major factor, considering its reported prevalence of 16% among LS patients with EC from our population.^50^ Furthermore, we relied on sequence data combined with database and in silico information but did not generate functional data to distinguish truly deleterious from harmless variants, which is another limitation of this study. For occasional genes (e.g., *ARID1A* and *TP53*), we did have the possibility to compare our sequencing results to immunohistochemical data from our previous studies,^16^, ^22^ and the datasets revealed a good overall concordance.

In summary, our investigation provides new information on key molecular steps that may lead to EC and/or OC in LS. When compared to knowledge available on corresponding sporadic cases,^15^, ^41^, ^44^, ^45^ many molecular features seem universal, including the nature of top mutant genes (Figure 3), similarity of endometrial hyperplasia to paired EC or OC (Figure 3 and Table 1), and the occurrence of cancer‐associated somatic variants in histologically NE, in the LS context years before cancer development (Figures 2A,B and S1). LS‐associated EC and OC tend to have indolent courses, with 10‐year survival of 98% and 89%, respectively,^3^ and the favorable prognosis seems attributable to inherent biological properties rather than, for example, less aggressive histological subtypes.^51^ In part reflecting the indolent behavior, no universally accepted guidelines for gynecological cancer surveillance in LS presently exist, since unequivocal evidence of clinical benefits is lacking.^52^ While reliable interpretations of the biological or clinical significance of somatic variants present in non‐neoplastic and preneoplastic tissues prior to cancer development await prospective long‐term follow‐up investigations, variant sharing between consecutive samples may point to a role in tumorigenesis. Pending confirmation by additional investigations, persistent molecular alterations in endometrial biopsy specimens might be a factor to consider more intensive surveillance or prophylactic hysterectomy in LS carriers.

## AUTHOR CONTRIBUTIONS

**Anni K. Kauppinen:** Conceptualization; data curation; formal analysis; visualization; writing – original draft; methodology; investigation; writing – review and editing; software; validation. **Alisa P. Olkinuora:** Conceptualization; formal analysis; methodology; investigation; supervision; writing – review and editing; software. **Jukka‐Pekka Mecklin:** Investigation; project administration; funding acquisition; resources. **Päivi T. Peltomäki:** Conceptualization; formal analysis; writing – original draft; investigation; supervision; project administration; writing – review and editing; funding acquisition.

## FUNDING INFORMATION

This work was funded by the Cancer Foundation Finland (to Anni K. Kauppinen, Alisa P. Olkinuora, and Päivi T. Peltomäki); Biomedicum Helsinki Foundation (to Alisa P. Olkinuora); the Jane and Aatos Erkko Foundation (to Jukka‐Pekka Mecklin and Päivi T. Peltomäki); the Academy of Finland (grant number 330606 to Päivi T. Peltomäki); and the Sigrid Juselius Foundation (to Päivi T. Peltomäki). The University of Helsinki Doctoral Programme in Biomedicine offered a paid doctoral student position to Alisa P. Olkinuora.

## CONFLICT OF INTEREST STATEMENT

Päivi T. Peltomäki reports a position in the Clinical Advisory Board of Lynsight Ltd. The other authors declare no conflict of interest.

## ETHICS STATEMENT

This study was approved by the Institutional Review Boards of the Departments of Obstetrics and Gynecology (040/95) and Surgery (466/E6/01) of the Helsinki University Central Hospital (Helsinki, Finland), and that of the Jyväskylä Central Hospital (Jyväskylä, Finland) (Dnro 5/2007). Collection of archival samples was approved by the National Authority for Medicolegal Affairs (Dnro 1272/04/044/07).

## Supporting information


**Table S1.** PanCancer panel design.

**Table S2.** Performance characteristics of the study specimens.

**Table S3.** COSMIC genes per cancer type considered in this study.

**Table S4.** Patient, sample and variant details of our study series.

**Table S5.** All non‐synonymous somatic variants by VarScan2 (*p* < .01) against blood (or normal endometrium if blood was unavailable).

**Data S1.** Supporting Information.

## Data Availability

The data that support the findings of this study are available from the corresponding author upon reasonable request.
