# Supplementary material for: Molecular analysis of the evolutionary history of endometrial and ovarian carcinoma in Lynch syndrome
Source: Int J Cancer. 2025 Aug 7;157(11):2294–308. doi: 10.1002/ijc.70074 (PMC12496004; doi:10.1002/ijc.70074)
Supplement: Supplementary file 6 — Data S1. Supporting Information. [file IJC-157-2294-s005.pdf]

Molecular analysis of the evolutionary history of endometrial and ovarian carcinoma in Lynch syndrome

Anni K. Kauppinen, Alisa P. Olkinuora, Jukka-Pekka Mecklin, Päivi T. Peltomäki

Contents

Supplement Table 1. Available in separate file. 2
Supplement Table 2. Available in separate file. 2
Supplement Table 3. Available in separate file. 2
Supplement Table 4. Available in separate file. 2
Supplement Table 5. Available in separate file. 2
Supplement Figure 1. 3
Supplement Figure 2. 4

Supplement Table 1. Available in separate file. Pan Cancer panel design.

Supplement Table 2. Available in separate file. Performance characteristics of the study specimens.

Supplement Table 3. Available in separate file. COSMIC genes per cancer type considered in this study.

Supplement Table 4. Available in separate file. Patient, sample, and variant details of our study series.

Supplement Table 5. Available in separate file. All nonsynonymous somatic variants by VarScan2 ($p < 0.01$) against blood (or normal endometrium if blood was unavailable).

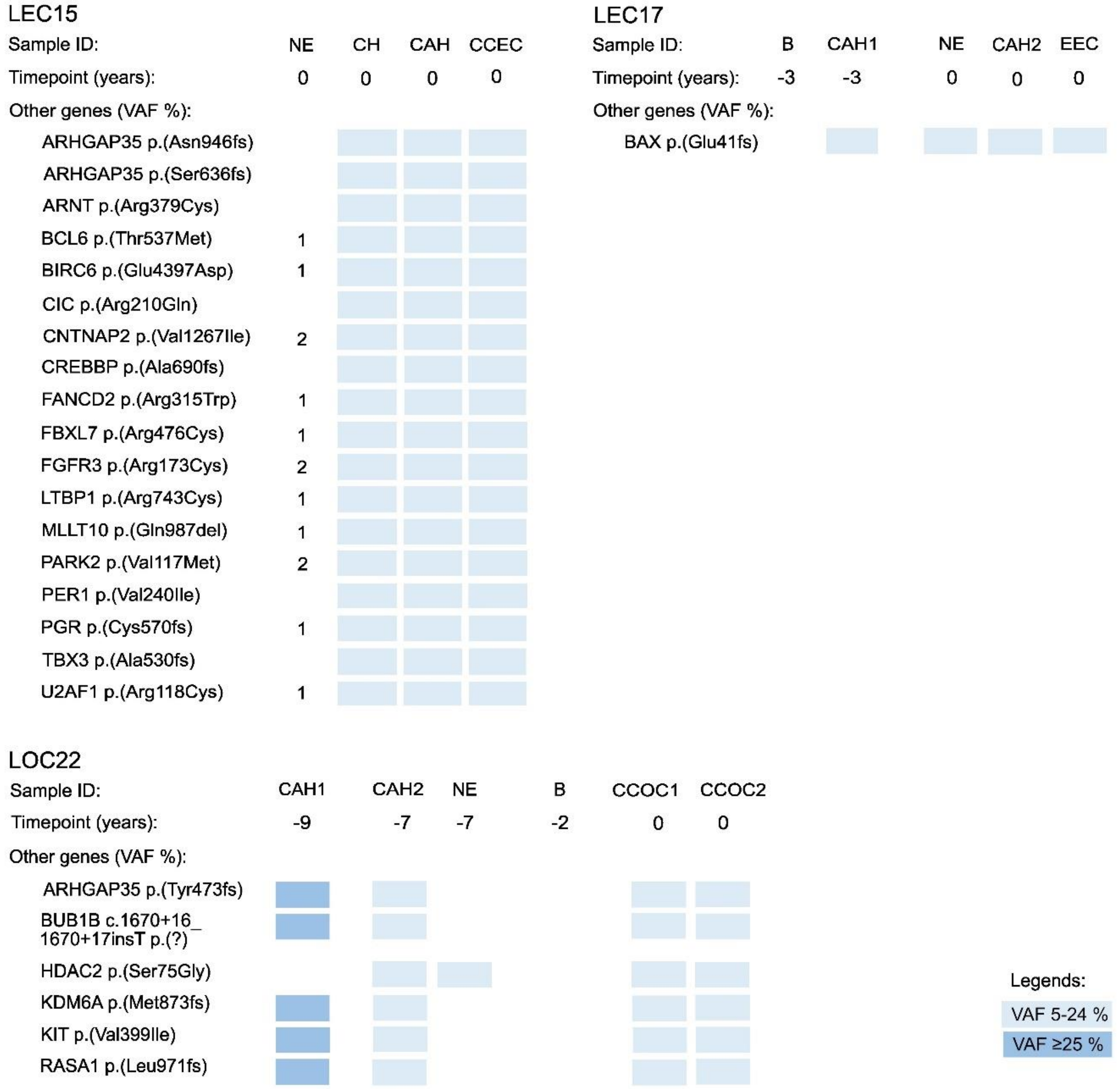

Supplement Figure 1. Chronological diagrams of LS cases that showed shared somatic variants in non-COSMIC genes, in addition to COSMIC genes. Variant sharing between carcinoma and two endometrial hyperplasia samples was required. Please see legend to Figure 2 for other specifications.

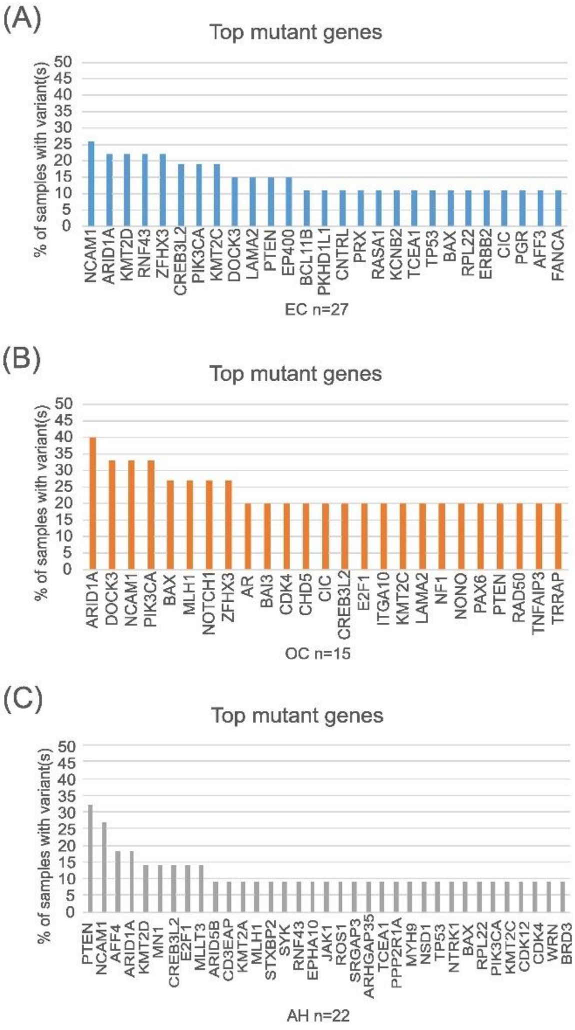

Supplement Figure 2. Genes having somatic variants with VAFs above 25% in over 10% of ECs (A), in at least 20% of OCs (B), and in at least 9% of atypical hyperplasias (C).
